# Editorial: The Role of Saliva in Arthropod-Host-Pathogen Relationships

**DOI:** 10.3389/fcimb.2020.630626

**Published:** 2021-01-28

**Authors:** Lucas Tirloni, Eric Calvo, Satoru Konnai, Itabajara da Silva Vaz

**Affiliations:** ^1^ Laboratory of Bacteriology, Tick-Pathogen Transmission Unit, Division of Intramural Research, National Institute of Allergy and Infectious Diseases, Hamilton, MT, United States; ^2^ Laboratory of Malaria and Vector Research, Molecular Entomology Unit, Division of Intramural Research, National Institute of Allergy and Infectious Diseases, Rockville, MD, United States; ^3^ Department of Disease Control, Faculty of Veterinary Medicine, Hokkaido University, Sapporo, Japan; ^4^ Centro de Biotecnologia, Universidade Federal do Rio Grande do Sul, Porto Alegre, Brazil; ^5^ Faculdade de Veterinária, Universidade Federal do Rio Grande do Sul, Porto Alegre, Brazil

**Keywords:** arthropod, saliva, host, pathogen, relationship

## Introduction

Hematophagous arthropods can transmit various pathogens causing diseases to humans, including malaria, Chagas disease, filariasis, leishmaniosis, dengue, Lyme disease, anaplasmosis, babesiosis, among other (WHO, 2020). In addition, these arthropods are vector of disease-causing pathogens to animals generating huge economic losses in livestock.

Saliva was a central component for the adaptation of the hematophagy in blood feeding arthropods and this habit evolved independently in many arthropod orders or even within insect families (Ribeiro, 1995). Saliva has a potent pharmacologically activity that interfere in the hemostatic and immune responses of vertebrate host (Ribeiro, 1995; Francischetti, 2009; Šimo et al., 2017) and pathogen transmission. Additionally, active compounds in arthropod saliva are potential useful as therapeutic tools (Chmelar et al., 2019). The study described by Li et al. shed light on comparative proteomic of a different species of blood feeding parasite, the parasitic isopod *Tachaea chinensis*, a parasite of shrimps. Similar to blood feeding arthropods feeding on mammals, there is evidence that isopod parasites may also inject anticoagulants or other compounds directly into the host to modulate host’s hemostasis and immune response. In this study, authors used a tandem mass tag-based quantitative proteomic approach to perform a comparative analysis between unfed and fed individuals, identifying 37 upregulated and 92 downregulated proteins in unfed *T. chinensis*, suggesting that organism’s energetic demand is increased during the search for a host. Similar to other hematophagous parasites, isopods may also employ biomolecules that affect host blood coagulation and defense systems. Differentially expressed proteins related to blood feeding identified in this study also were described in the saliva of other hematophagous arthropods (Mans, 2011; Tirloni et al., 2014; Chmelař et al., 2016).

The protein composition of the saliva of hematophagous arthropods and the correlation with expression in salivary glands are essential to understand the feeding process and immunomodulation of host defenses (Tirloni et al., 2017; Antunes et al., 2019). Technological advances, including large-scale DNA sequencing and proteomic analysis, have increased the identification of genes and proteins, supporting our comprehension governing vector-host-pathogen interactions. In a fascinating review, Mans performs a historical analysis and reflection of the development of methods used to study and identify the protein composition related to the tick-host interface. This work summarizes the advances made over the years to understand and describe the complexity present within this interface. The high-throughput *in silico* analyses are widening rapidly and the development of new algorithms are increasing the accuracy of analyses and the importance for biological sciences (Hernández-Vargas et al., 2017; Bensaoud et al., 2019; Jia et al., 2020; Polanska et al., 2020; Tirloni et al., 2020). Recent advances in tick transcriptomic and proteomic studies have revealed over thousands of different transcripts coding for proteins in different tick species (Karim et al., 2011; Schwarz et al., 2013; Chmelař et al., 2016; Tirloni et al., 2020). As more and more studies contribute to our knowledge of tick genes and proteins, it becomes increasingly important to develop new approaches to help the annotation and classification of identified sequences. A tick-specific protein database is import since previous studies showed the presence of protein families that are found exclusively in ticks. TickSialoFam (TSFam) is a database aimed to assemble a curated collection of salivary genes and proteins in addition to improve the annotation of their putative functions. The initial version of the database identified 136 tick salivary secreted protein families. Moreover, with the increasing availability of whole-genome sequencing data, the TSFam database can be updated to include these new identified sequences (Ribeiro et al.).

Quantification of the cellular activity in different conditions or times, such as the parasite relationship, is instrumental to understand the cell or organ functions and susceptibility to diseases and several strategies have been developed for the quantification of proteins using mass spectrometry (Van De Merbel, 2019). This approach was used to compare proteins in the saliva of five vector species of the Chaga`s disease pathogen, *Trypanosoma cruzi*, including *Triatoma infestans*, *Triatoma dimidiata*, *Dipetalogaster maxima*, *Rhodnius prolixus*, and *Rhodnius neglectus* (Santiago et al.). Data showed similarities and differences in protein profile in saliva of these species. A notable observation was the presence of unique proteins for each triatomine species that could be useful as marker for species identification.

Another interesting study published in this issue provided evidence that a salivary protein affect vitellogenin uptake in the ovary and thereby play a role in the modulation of tick reproduction. The RH36 is an immunosuppressant protein that regulate the host immune system during tick feeding. Homologous proteins to RH36 were identified in other ticks (Aljamali et al., 2009; Anatriello et al., 2010) and characterized as immunosuppressant molecule, including in *Dermacentor andersoni* (Bergman et al., 1998) and *Haemaphysalis longicornis* (Konnai et al., 2009). Surprisingly, RNAi-mediated gene silencing of RH36 induces a reduction in tick oviposition and also affects HSP70 expression in the immature ovary of engorged ticks. This raises the possibility that RH36 interferes in tick vitellogenin uptake and then control ovary cell maturation by modulating HSP70 expression, and controlling tick oviposition (Wang et al.).

As mentioned, saliva facilitates establishment of infection and increases transmission in the vertebrate host. However, host susceptibility to parasite infection also is markedly affected by genetic differences. Many studies have shown that susceptibility to parasites is influenced by genetic characteristics of both host and pathogen. In addition, there are solid evidences for the existence of host genetic component controlling expression of parasite proteins (Popara et al., 2013; Tirloni et al., 2017). A perfect example is that *Bos taurus* are more susceptible to *Rhipicephalus microplus* infestations than *Bos indicus* (Garcia et al., 2020). The characterization of tick salivary gland gene expression in tick-susceptible and tick-resistant hosts can be important for the identification of potential targets for the development of new control methods. Giachetto et al. study identified 137 sequences as differentially expressed genes between ticks feeding on tick-susceptible or tick-resistant cattle.

Bioinformatics analysis associated to serological data are useful strategies for antigen selection in vaccine development. Sera from rabbits repeatedly infested with *Ixodes ricinus* were used to identify salivary immunogenic antigens. Using this approach, Perner et al. identified metalloproteases essential to tick initial feeding and engorgement, suggesting immunomodulatory or anti-hemostatic properties of these enzymes. To corroborate the role of metalloproteases at the tick/host interface, authors fed ticks micro-injected with a zinc metalloprotease inhibitor, which impacted tick feeding. A proteomic approach was also used to identify potential vector exposure markers (Zeyrek et al., 2011) in mosquitoes. The global spread of the mosquito *Aedes albopictus* increases the risk and burden of *Aedes*-transmitted viruses to temperate areas, highlighting the need to improve vector surveillance methods. With a proteomic approach, Montero et al. identified a correlation between antibodies against an *A. albopictus* salivary gland protein and exposure to mosquito bites. A similar strategy was used by Londono-Renteria et al. to identify a positive correlation among the antibodies against an *Anopheles darlingi* salivary gland antigen (apyrase) and antibodies against the *Plasmodium vivax* and *P. falciparum* antigens in patients infected with malaria. Individuals with high IgG levels are five times more likely to have malaria infection than uninfected persons. Both studies provide strong evidences to use salivary proteins in tools for monitoring the human-mosquito exposure.

Similarly, with the objective to develop diagnostic and research tools Contreras et al. characterized the use the zebrafish as a new animal model to study of allergic reactions and relation among the immune mechanisms in response against the α-gal epitope (Galα1-3Galβ1-(3)4GlcNAc-R) presents in tick saliva and red meat consumption. With the development of new techniques, Hermance et al. used RNA *in situ* hybridization to analyze the cellular localization of Powassan virus at the *Ixodes scapularis* feeding site. Furthermore, this methodology can be used in to identify virus replication in tissues of different mammalian hosts and tick vectors.

Scientific and clinical interest in parasite-derived molecules and their immunomodulatory properties is partially focused in the development of novel drugs for treating diseases. The immunomodulatory effect of the mosquito saliva is reviewed by Guerrero et al. showing the strongly effect in the transmission and the establishment of pathogens in the host. The focus is on the role of saliva in arboviruses transmission and the potential use of salivary proteins for the control of pathogen transmission through the development of effective vaccines. Sumova et al. showed the immunomodulatory properties of three *Phlebotomus perniciosus* salivary proteins. These proteins inhibited macrophages nitric oxide production and rSP03 proteins increased IL-10 and decreased TNF-α secretions. This data helps understanding the immunomodulatory role of saliva and its participation in *Leishmania* transmission. Similarly, the immunomodulatory effect of parasite saliva also has an effect on pathogen transmission to the vertebrate host (Titus and Ribeiro, 1988; Šimo et al., 2017; Aounallah et al., 2020). Based on *in vitro* experiments with BHK cells expressing a salivary peroxiredoxin from *Haemaphysalis longicornis*, Kusakisako and colleagues demonstrate that this protein facilitates the replication of the tick-borne encephalitis virus by some yet unknown mechanism (Kusakisako et al.).

In conclusion, this Research Topic shed light on the roles of arthropod saliva facilitating blood meal acquisition and pathogen transmission. Moreover, different rationale strategies to develop and improve vaccine efficacy and diagnostic tools were pursued. A better understanding in this field is fundamental to the discovery and implementation of control and prevention strategies, including vaccines, not only against specific pathogens but also the arthropod itself.

## Author Contributions

All authors listed have made a substantial, direct, and intellectual contribution to the work and approved it for publication.

## Funding

LT and EC are supported by the Intramural Research Program of the National Institute of Allergy and Infectious Diseases, National Institutes of Health, USA. SK is supported by grants from JSPS KAKENHI, AMED, and JSPS (Japan), and IV is supported by grants from CAPES and CNPq (Brazil).

## Conflict of Interest

The authors declare that the research was conducted in the absence of any commercial or financial relationships that could be construed as a potential conflict of interest.
